# Numerical Modeling and Optimization of Large-Scale Molten Titanium Levitation

**DOI:** 10.3390/ma18061268

**Published:** 2025-03-13

**Authors:** Sławomir Golak, Jakub Wyciślik, Radosław Zybała, Robert Hanusek

**Affiliations:** 1 Department of Industrial Informatics, Faculty of Materials Engineering, Silesian University of Technology, Krasińskiego 8, 40-019 Katowice, Poland; jakub.wycislik@polsl.pl (J.W.); radoslaw.zybala@polsl.pl (R.Z.); robert.hanusek@alorybnik.polsl.pl (R.H.); 2 Łukasiewicz Research Network—Institute of Non-Ferrous Metals, Sowińskiego 5, 44-100 Gliwice, Poland

**Keywords:** reactive metals, titanium, levitation melting, magnetohydrodynamics (MHD), numerical modeling, numerical optimization

## Abstract

Melting reactive metals and alloys, such as titanium, poses a significant challenge due to the risk of crucible damage and contamination of the molten material. Full levitation melting presents a promising solution; however, its application has largely been limited to small laboratory samples. This paper introduces a methodology for modeling (in a 2D axisymmetric domain) and optimizing a new large-scale levitation melting system and demonstrates its application to pure titanium. The system features a torus-shaped load positioned within a gutter-shaped coil. Numerical experiments using this approach confirm the feasibility of stable levitation for a substantial mass (2.6 kg) within a newly designed electromagnetic levitation system.

## 1. Introduction

Reactive metals (e.g., titanium, tantalum, niobium, and molybdenum) have many potential applications in modern industries such as automotive, aviation, and aerospace. However, processing them in the molten state presents a serious problem. The use of vacuum in classic induction melting (VIM) devices [1,2,3] does not solve this problem. Contact of molten metal with the crucible material leads to the damage of the device and, above all, unacceptable contamination of the processed metal [4,5,6,7,8,9]. Therefore, despite their good energy efficiency, these devices can only be used to a very limited extent for the processing of reactive metals. The solution to this problem is levitation melting, either in its semi-levitation [10,11,12,13] or full levitation form [14,15,16,17,18,19]. In this process, an alternating electromagnetic field is used to melt and heat the metal (by generating Joule heat within it) and to partially or fully support the liquid metal as a result of Lorentz forces.

So far, only the semi-levitation version has found industrial application. It uses a water-cooled metal cold crucible, which supports the molten metal from below. Due to the cooling process, a solidified metal layer (known as a skull) forms, preventing direct contact between the reactive liquid metal and the crucible material [20,21,22,23,24,25]. This solution allows for the melting of even multi-kilogram loads, but despite continuous development [26,27,28], it still has some drawbacks. The need to remove heat from the molten metal to form the skull results in heat loss and, therefore, low energy efficiency of the device. In addition, a major problem caused by continuous heat dissipation is the difficulty in achieving the desired superheat of the processed metal. Furthermore, despite the eventual separation of the molten metal from the cold crucible by the skull, there is a risk of metal load contamination during skull formation, which can be critical in applications requiring very high purity.

Full levitation melting does not have these disadvantages (low energy efficiency and risk of metal contamination). However, despite being invented in the 1920s, it has not yet found industrial applications due to the limitation of melt mass to only a few grams. The reason is the use of various vertical coil variants in this process, in the axis of which the processed metal is placed. In such coils, the applied alternating electromagnetic field disappears in their axis, which causes the lack of support by Lorentz forces of the molten metal in this area [29]. For small laboratory-scale samples, surface tension prevents metal leakage in this region. However, as the metal mass increases, its hydrostatic pressure rises, eventually overcoming surface tension forces and causing the metal to leak downward. One approach to addressing this issue is the use of a longitudinal electromagnetic levitator [30]. While this solution has the potential to increase the load mass, it remains constrained by the loss of supporting electromagnetic forces at the center of the load.

Some interesting proposals exist for load stabilization through automatic, continuous process control based on vision monitoring and a parallel numerical model used in this control [31]. However, these methods do not enable a fundamental breakthrough in overcoming the mass barrier.

In recent years, however, solutions have emerged that enable significantly exceeding the few-gram limit for the full levitation process. The first solution [32,33,34] is based on complete change of the electromagnetic field orientation from vertical to horizontal which was aimed at eliminating the above-mentioned problem of the lack of load support in the area of vertical axis of the system. Although, the proposed solution breaks the stagnation in the development of levitation melting, it has its two important drawbacks. First, it requires the use of a complex dual-frequency power supply system that can limit the up-scaling of the solution. Additionally, due to the use of a rotating magnetic field, some complications may occur with maintaining the stability of the load during melting. The second solution is based on the standard quasi-static, vertically oriented electromagnetic field. The idea is to change the shape of a metal load into a toroid placed in a gutter-shaped coil. The paper [29] presenting the solution contains only a discussion of the concept of this solution confirmed only by electromagnetic calculations for a metal with an assumed constant and unreal shape.

Table 1 presents a comparison of the features of the discussed methods for melting reactive metals. As shown, large-scale levitation melting exhibits all four advantages at least at a ‘high’ level. This suggests that, for melting reactive metals, this method has no apparent disadvantages compared to existing methods, thereby justifying further research into its development.

The mentioned paper [29] does not determine whether it is possible to select the coil geometry and power supply parameters in such a way that stable levitation of liquid metal (i.e., shape-changing) can be achieved. The confirmation that a large mass of liquid metal can be maintained in levitation is based on designing the coil geometry and power supply parameters to ensure the stable positioning of the molten metal at the center of the coil. The paper proposes a coil geometry that initially supports the load from below and prevents it from escaping to the sides. However, the stable, desired position of the load is ultimately determined by the values of the currents supplying four separate coil sections. To determine these currents, a methodology for optimizing the levitation process had to be developed. The target optimization criterion was established using a numerical model that coupled electromagnetic and hydrodynamic fields. Performing a full numerical simulation of levitation up to the point of load stabilization takes several hours. Relying solely on this criterion for optimization would be too time-consuming. Therefore, in the proposed hierarchical optimization methodology, a surrogate criterion based solely on the electromagnetic model was employed, significantly reducing the total time required to obtain a solution. Nevertheless, the full coupled model is still integrated into the optimization process, enabling the analysis of flows in a stabilized metal load and providing data that cannot be obtained through measurement methods.

## 2. Materials and Methods

### 2.1. Research Object

**Figure 1:**
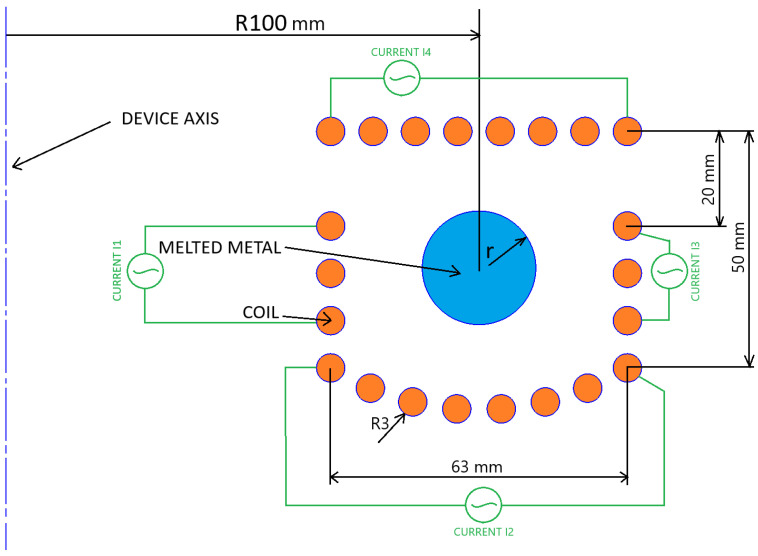
Schematic diagram of a large-scale levitation melting system (**R**—the main radius of the toroidal load, **r**—its cross-section radius).

The feasibility studies of the process were conducted for a torus-shaped titanium load with a main radius of 100 mm. The metal was levitated in an axially symmetric coil with a cross-section shown in Figure 1. The coil cross-section was designed to provide support for the load from below and prevent the load from escaping to the sides. The top section of the coil is supplied with a current that is in the opposite phase (shifted by π) to the currents supplying the rest of the coil. Although the cross-section shown in the figure is symmetrical, it is not symmetrical in the global system because the device’s axis lies outside the coil area. As a result, the outer part of the coil (farther from the axis) has a larger surface area than the corresponding inner part. This would cause, with the same current flowing through each turn, a greater intensity of the electromagnetic field on the right side of the load, and thus greater Lorentz forces on this side pushing the metal towards the axis of the system. Therefore, it is necessary to separately power the 4 sections of the coil with different currents, and the current intensities must be selected so that the Lorentz forces balance the force of gravity and at the same time keep the metal (the torus cross-section) in the center of the coil. All coil sections were powered with a current at a frequency of 10 kHz. The titanium properties assumed in the numerical model are given in Table 2.

### 2.2. Numerical Model

Due to the device’s geometry, an axisymmetric 2D model can be used to represent it. This simplification significantly accelerates the calculations performed repeatedly during the optimization process. The model was implemented based on a methodology experimentally validated for classical levitation melting [35].

**Figure 2:**
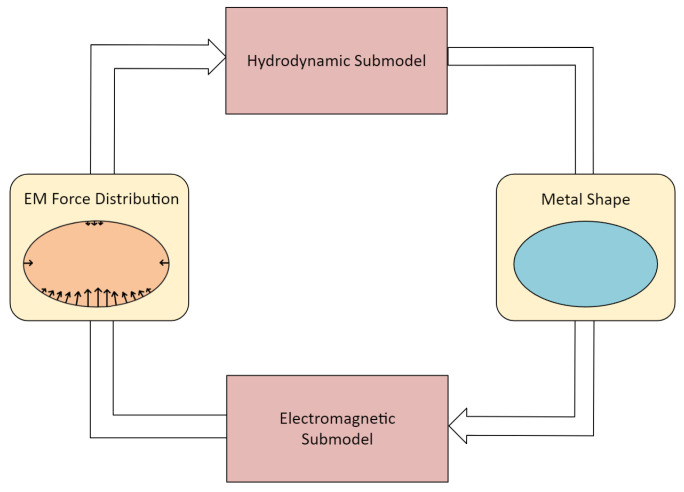
Schematic diagram of the full model of the levitation process, including the coupling of the hydrodynamic (master) submodel and the electromagnetic (slave) submodel.

The full model of the levitation melting process must include the coupling of electromagnetic and hydrodynamic fields (Figure 2).

The electromagnetic submodel calculates the distribution of the electromagnetic field and, more importantly, the Lorentz forces that support the levitating metal and alter its shape. The hydrodynamic submodel determines the metal’s flow and the shape changes induced by electromagnetic forces. It is important to consider that changes in the shape of the liquid metal affect the distribution of the electromagnetic field, which in turn alters the position of the load equilibrium point for both gravity and electromagnetic forces. Reciprocally, the Lorentz forces generated by this field change the shape of the metal. Therefore, both fields (hydrodynamic HD and electromagnetic EM) must be coupled in this regard during the process simulation. The hydrodynamic simulation plays the primary role in standard methodologies [35,36,37,38,39,40]. At each time step, the current shape of the metal is determined and transferred to the electromagnetic submodel, which then calculates and returns the distribution of Lorentz forces acting on the liquid metal. These forces modify the shape of the metal load, and the simulation cycle starts again.

#### 2.2.1. Electromagnetic Submodel

The electromagnetic problem was solved using finite element harmonic analysis at a frequency of 10 kHz. For the numerical domain, the magnetic vector potential equation was applied in the following form:

(1) $$\nabla \times \left(\frac{1}{\mu }\nabla \times A\right)+j\omega \sigma A=J_{s}$$

where μ is the magnetic permeability, σ is the electric conductivity, ω is the angular frequency, and $J_{s}$ is the current density source.

Knowing the distribution of the magnetic vector potential A, both the magnetic induction B and the eddy current density J can be determined as follows:

(2) B=∇×A,

(3) J=−jωσA.

With the current density and the induction, one can obtain the volume density of the Lorentz force acting on the molten metal:

(4) $$f_{L}=\frac{1}{2}Re\left(J\times B^{*}\right)$$

where $B^{*}$ is the complex conjugate of B.

**Figure 3:**
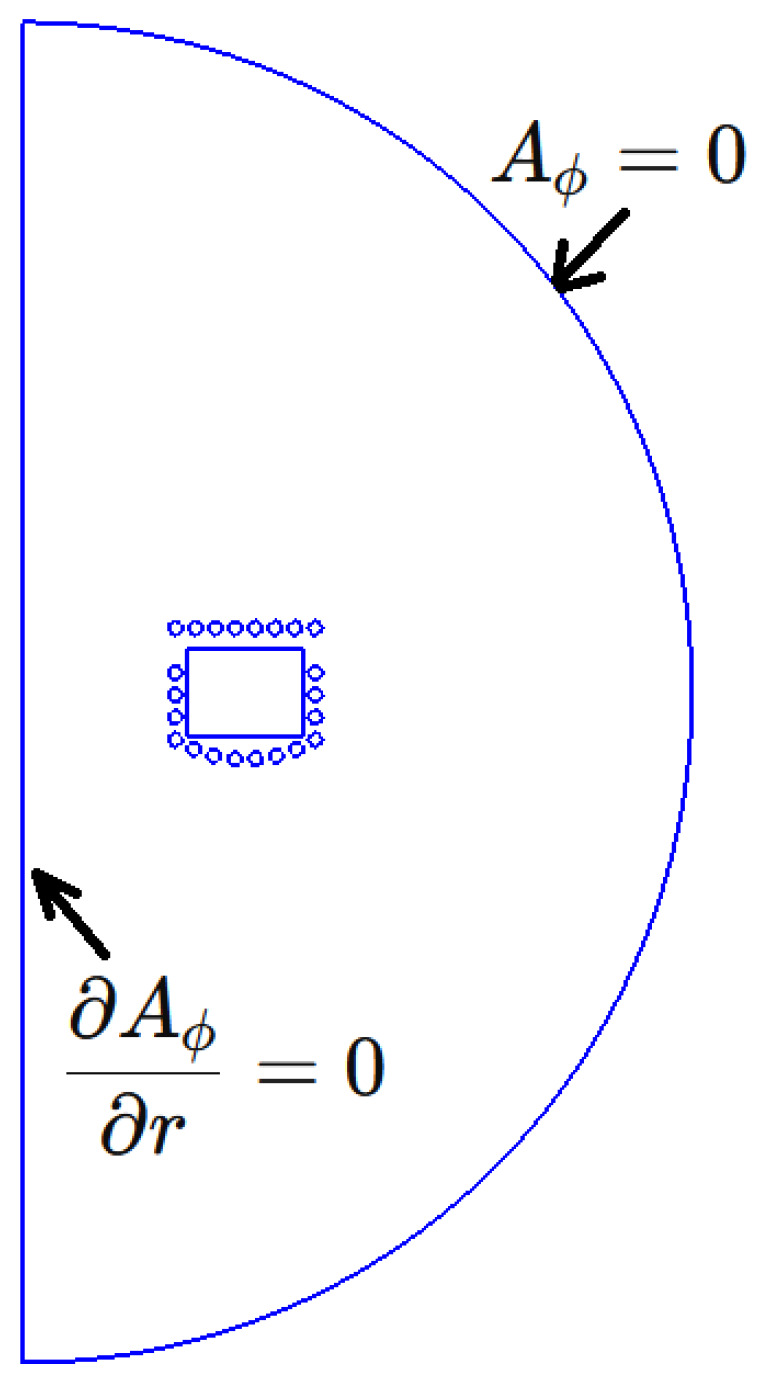
Boundary conditions for the electromagnetic submodel. $A_{\Phi }$ is the azimuthal magnetic vector potential and *r* is the radial coordinate.

Figure 3 shows the boundary conditions for the electromagnetic submodel. For the 2D problem in an axisymmetric system, only the azimuthal component is considered. At the domain boundary, the vector magnetic potential associated with the electric field must be zero. Additionally, due to the symmetry of the problem, the field variables must remain continuous along the axis.

The electromagnetic submodel was implemented based on the GetDP software 3.5.0 [41,42].

#### 2.2.2. Hydrodynamic Submodel

The hydrodynamic model is based on the Navier–Stokes momentum conservation Equation (5). Air and metal are modeled as incompressible fluids. One of the source terms is the volume density of the Lorentz force $f_{L}$ acting on the liquid metal, provided by the electromagnetic model:

(5) $$\frac{\partial }{\partial t}\left(\rho v\right)+\nabla \cdot \left(\rho vv\right)=-\nabla p+\nabla \cdot \left[\eta_{eff}\left(\nabla v+\nabla v^{T}\right)\right]+f_{L}+f_{S}+\rho g$$

where ρ is the fluid density, v is the fluid velocity, *p* is the pressure, $\eta_{eff}$ is the effective viscosity determined from the k-ω turbulence model, $f_{S}$ is the surface tension force, and g is the gravity.

The k-ω turbulence model used is highly efficient, adding only two additional differential equations to the system—an essential factor in maintaining computational efficiency. Furthermore, it performs well at low Reynolds numbers, enabling the reproduction of laminar flow conditions when necessary.

The flow within the levitation system was modeled as a multiphase system with a free surface. To simulate this process, the Volume of Fluid (VOF) method [43] was employed. This method is a standard solution for free-surface MHD problems, where the metal shape is transferred to the electromagnetic model via the conductivity distribution, as it operates based on the volume fraction of the metal. To track the evolution of the molten metal’s free surface, the volume fraction conservation equation for the metal phase was solved:

(6) $$\frac{\partial }{\partial t}\left(\alpha_{m}\rho_{m}\right)+\nabla \cdot \left(\alpha_{m}\rho_{m}v\right)=0$$

where $\rho_{m}$ is the metal density. The air phase fraction $\alpha_{a}$ (for the considered two-phase system) is calculated from the equation:

(7) $$\alpha_{a}=1-\alpha_{m}.$$

In the VOF approach, material properties ρ and $\eta_{eff}$ which appear in (5) were determined based on the weighted average of volume fraction and property of air and metal phase.

The surface tension force (additional source term $f_{S}$ in Equation (5)) is determined based on a reconstructed shape of the interface between the liquid metal and air, corresponding to the current phase fraction distribution.

**Figure 4:**
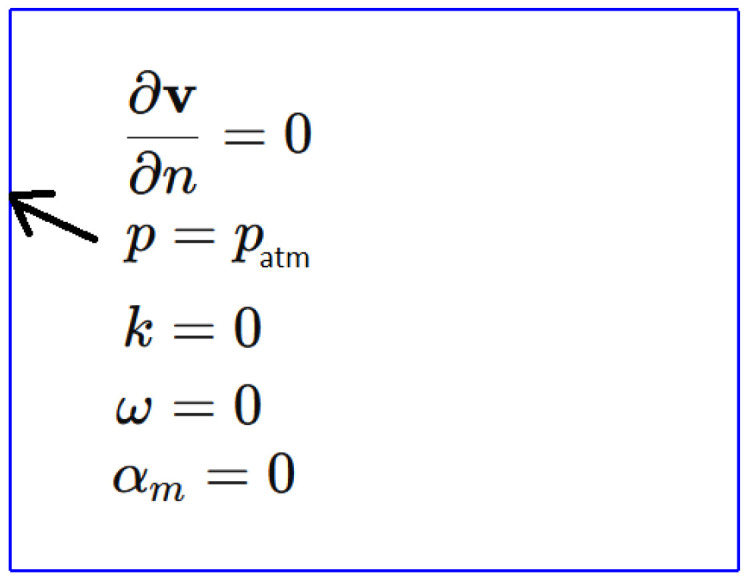
Boundary conditions for the hydrodynamic submodel.

Figure 4 shows the boundary conditions defined for the hydrodynamic submodel. It is assumed that the velocity remains unaffected and that atmospheric pressure is applied at the boundary of the computational domain. Furthermore, turbulence is assumed to be absent at the boundary of this region.

The hydrodynamic submodel was implemented based on Ansys Fluent software (https://www.ansys.com/products/fluids/ansys-fluent, accessed on 9 March 2025).

#### 2.2.3. Computational Mesh

In the electromagnetic and hydrodynamic submodels, a coherent mesh was used for the region where metal might be present, forming a two-phase system with a free surface. Since the electromagnetic Lorentz forces influence both the process dynamics and the shape and position of the metal, the initial mesh size was determined based on the penetration depth of the electromagnetic field at the applied frequency, as well as the metal’s electrical conductivity and magnetic permeability (Table 2). The calculated penetration depth, using the formula provided in Equation (8), is 0.0067 m:

(8) $$\delta =\frac{1}{\sqrt{\pi f\mu \sigma }}$$

where *f* is the electromagnetic field frequency.

To accurately capture the key effects of the electromagnetic field at the free metal surface, the mesh size in this region was set to an order of magnitude smaller than the penetration depth—0.0005 m.

### 2.3. Optimization

**Figure 5:**
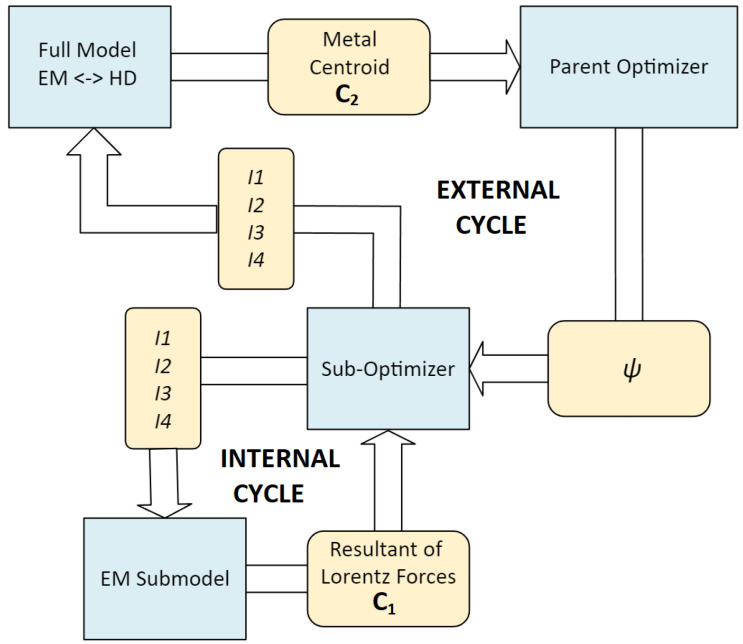
Diagram of a hierarchical optimization system containing an internal cycle (obtaining force balance) and an external cycle (obtaining the desired load position).

The optimization of the current values driving the 4 coil sections was carried out for efficiency reasons in two cycles. The diagram in Figure 5 shows the developed hierarchical optimization system.

**Figure 6:**
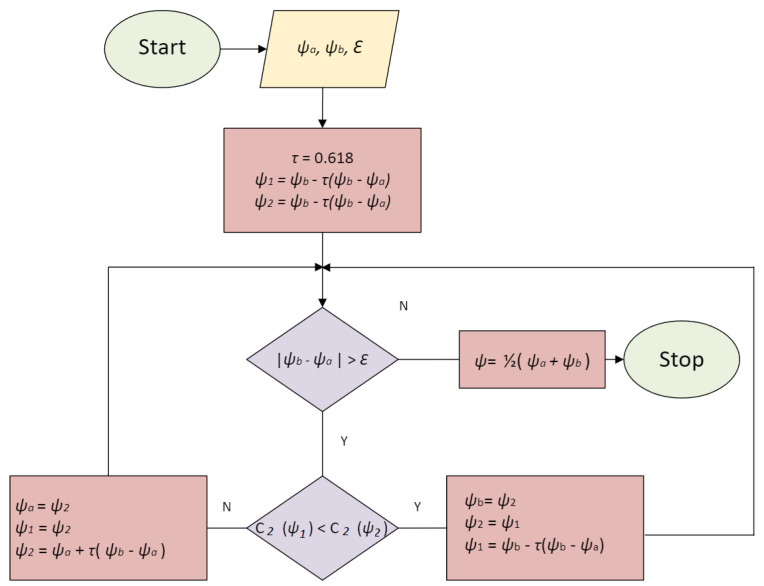
Schematic diagram of the algorithm (based on the golden-section search method) for the external optimization cycle allowing to determine the ψ correction value in the definition of criterion $C_{1}$ component $C_{r}$.

In the first, internal optimization was carried out for a constant position and an ideally circular cross-section of the load. This allowed to implement the optimization process based solely on the electromagnetic submodel and the efficient multidimensional optimization algorithm selected experimentally. The purpose of the internal optimization algorithm was to balance the forces (electromagnetic and gravitational) acting on the metal load. Since a 2D model is used here, the criterion determination takes a few seconds. For this reason, there is no need to replace the FEM model with more sophisticated surrogate models [30]. However, the constant position and geometry of the liquid load is an unrealistic case. In reality, the molten load undergoes deformation and associated displacement, which means that the current values determined for a constant position and geometry of the metal will not ensure the retention of the deformed metal in the center of the coil cross-section. Therefore, in the second, external cycle of current optimization, a one-dimensional optimization of the parameter correcting the resultant of horizontal forces acting on the load was performed. The purpose of this optimization cycle was to maintain a constant position of the load in the center of the coil cross-section. Determining the criterion for this optimization cycle required a simulation based on coupled electromagnetic and hydrodynamic submodels. However, due to the one-dimensionality of the optimization problem, a fast minimization algorithm based on the golden-section search could be used (Figure 6), which allowed determining the solution in an acceptable time despite the time-consuming criterion.

The assumption of the internal optimization cycle was to select the currents supplying the coil sections in such a way as to achieve a balance of forces acting on the levitated metal. The vertical component of the Lorentz force field should balance the gravitational force acting on the load:

(9) $$C_{a}=\int_{V}\rho \;g\;dV+\int_{V}\rho \;f_{\mathrm{LF}}\cdot {\hat{u}}_{a}\;dV=0$$

where *g* is gravity acceleration and ${\hat{u}}_{a}$ is the axial unit vector.

The horizontal component of the Lorentz force must balance the surface tension forces that tend to compress the torus toward its axis:

(10) $$C_{r}=\int_{V}\rho \;f_{\mathrm{LF}}\cdot {\hat{u}}_{r}\;dV+\psi =0$$

where the ψ value is a correction resulting from the change in the distribution and magnitude of electromagnetic forces and surface tension due to the deformation of the metal shape compared to the ideal circular cross-section assumed in the internal cycle of optimization.

Based on these equilibria, an internal optimization criterion can be defined:

(11) $$C_{1}=\sqrt{C_{a}^{2}+C_{r}^{2}}$$

The optimization parameters are the supply currents of the four coil sections $I_{1}$, $I_{2}$, $I_{3}$ and $I_{4}$. The constraint of the optimization is only the minimal value of these currents:

(12) $$I_{i}>100$$

The adoption of a minimum current value of 100 A for each of the four coil sections is intended to ensure the action of Lorentz forces on each side of the load and to prevent the metal from getting too close to the coil in the event of possible disturbances.

The parameter ψ present in the equation is subject to additional optimization in the external optimization cycle. Due to the one-dimensionality of the problem and the lack of local minima, the optimal value of ψ was determined using the golden-section search method (Figure 6). The optimization criterion is the maximum deviation of the horizontal position of the load in the time period from 150 (after adopting a stabilized shape) to 200 s:

(13) $$C_{2}=\max_{t\in [150,200]}\sqrt{{\left(x\left(t\right)-x_{d}\right)}^{2}+{\left(y\left(t\right)-y_{d}\right)}^{2}}$$

where $x_{d}$ and $y_{d}$ are the desired horizontal and vertical positions of the load. The load position was defined as the centroid of the metal cross-section:

(14) $$x=\frac{\sum_{i=1}^{N}\alpha_{m,i}x_{i}}{\sum_{i=1}^{N}\alpha_{m,i}}$$

(15) $$y=\frac{\sum_{i=1}^{N}\alpha_{m,i}y_{i}}{\sum_{i=1}^{N}\alpha_{m,i}}$$

where $\alpha_{m,i}$ is the volume fraction of metal in the i-th cell and $x_{i}$, $y_{i}$ are the coordinate of the cell centroid.

## 3. Results and Discussion

The tests were carried out for different titanium masses resulting from the initial cross-sectional radius of the metal load (12, 15, and 18 mm). For each mass of metal, the optimization of the currents supplying the four coil sections was performed based on the methodology described in Section 2.3.

**Figure 7:**
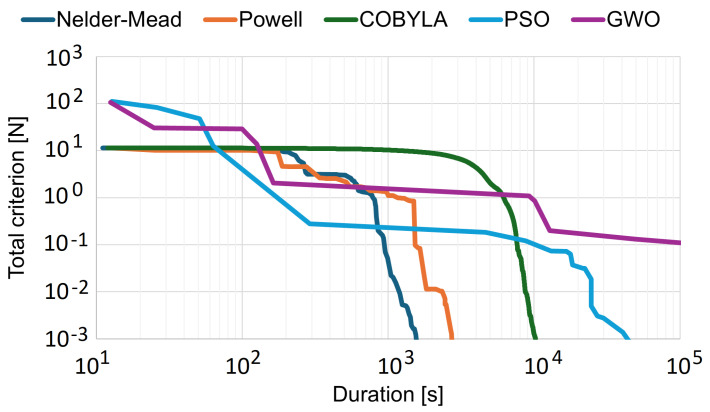
Example of progress during the internal optimization cycle, implemented using popular algorithms: Powell’s conjugate direction method, the downhill simplex method (Nelder–Mead), constrained optimization by linear approximation (COBYLA), particle swarm optimization (PSO), and the grey wolf optimizer (GWO). Real optimization times for Intel Core i7-7500U CPU 2.70 GHz.

Initial studies included the selection of the most efficient multidimensional optimization algorithm for the internal optimization cycle. Figure 7 shows the real-time progress of minimizing criterion $C_{1}$ (11) using different optimization algorithms. Three popular local algorithms (Powell’s conjugate direction method, the downhill simplex method, and constrained optimization by linear approximation), along with two example global algorithms were analyzed (particle swarm optimization and the grey wolf optimizer). Local algorithms, particularly the Nelder–Mead downhill simplex method (which was ultimately chosen), allowed obtaining a value of the force balance criterion close to zero in the shortest time because the physics of the process suggests a solution landscape devoid of local minima. Local algorithms effectively determine optimal solutions in systems where the criterion value is determined in the FEM model [44]. Global algorithms, despite their advantages, required significantly more time to converge. The time to obtain a solution was more than two orders of magnitude longer, which is unacceptable in this context. This conclusion based on only the example algorithms can be extended to the vast majority of algorithms in both groups.

Table 3 shows optimized values of the currents supplying sections of the coil. As can be seen, as the metal mass increases, the current in the lower coil increases to balance the increasing gravitational force. Simultaneously, the current in the upper part of the coil (with opposite phase), which is supposed to prevent the load from being thrown upwards, decreases. This is obvious because the force of gravity plays the same role. Interestingly, the currents feeding the side sections also decrease because, due to the increased size, the metal is closer to these sections.

**Figure 8:**
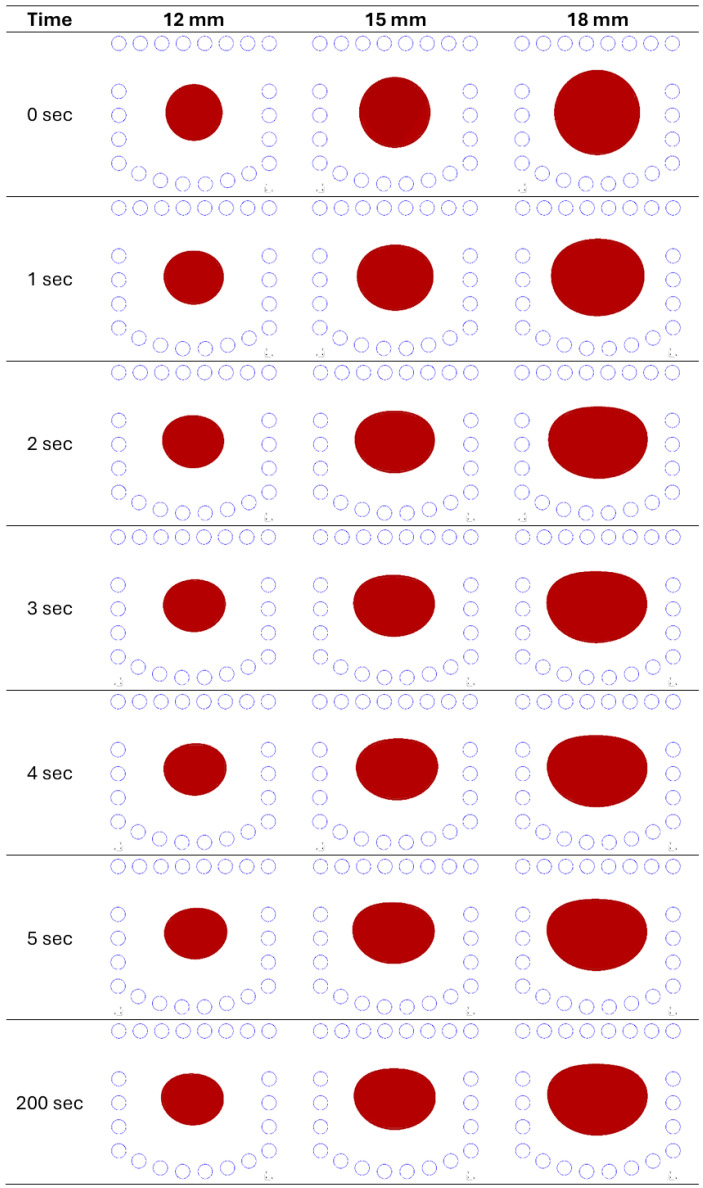
Evolution of the shape of the metal during the process (cross-section of a torus with radii of 12, 15, and 18 mm).

Based on the full model including the coupling of hydrodynamic and electromagnetic submodels, the simulation of the changes in the position and geometry of the load was performed until relative stabilization was achieved after 200 s of simulation time. The initial position and shape of the load were assumed to be consistent with the position for which the currents were optimized. Figure 8 shows the changes in the shape of loads of different sizes during the simulation.

**Figure 9:**
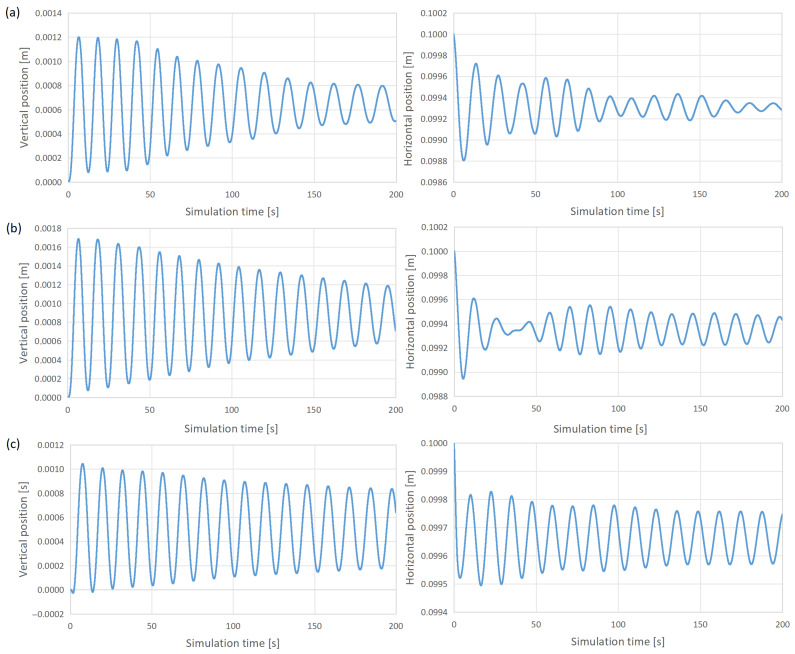
Horizontal and vertical load oscillations during simulation. Load cross-section radii: (**a**) 12 mm, (**b**) 15 mm, and (**c**) 18 mm.

In addition to changes in shape, which, according to Figure 8, quickly stabilize, the position of the load is subject to slowly damped oscillations. Figure 9 shows the horizontal and vertical positions of the centroid determined using Formulas (14) and (15). The larger load, the slower the oscillations decay, which is caused by the greater mass of the metal, but also by a greater change in shape compared to the initial shape (greater initial unbalance). A dynamic control of the coil currents can be used to dampen these oscillations [31]. However, since these oscillations are damped, it enables the prediction of the stability of the analyzed process on the current scale.

**Figure 10:**
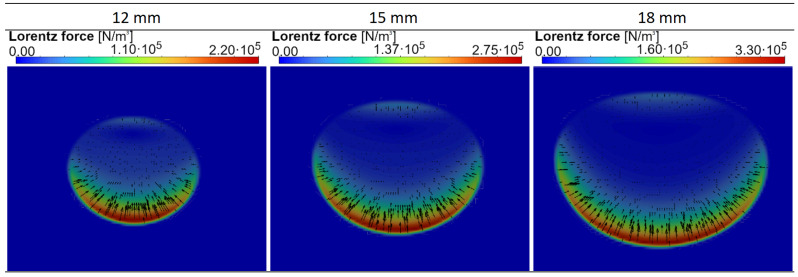
Volume density distribution of the Lorentz force supporting the metal after 200 s of the process for three sizes of the load (cross-section of a torus with radii of 12, 15 and 18 mm).

Figure 10 shows the volume density distribution of Lorentz forces supporting loads of different sizes. As can be seen, the idea of the new levitation device is confirmed here, the metal load over its entire width is supported from below by Lorentz forces, in contrast to classical levitation melting devices. From above can be seen the presence of forces blocking its upward movement. Thanks to optimization, the force distribution is more or less symmetrical in the horizontal direction.

**Figure 11:**
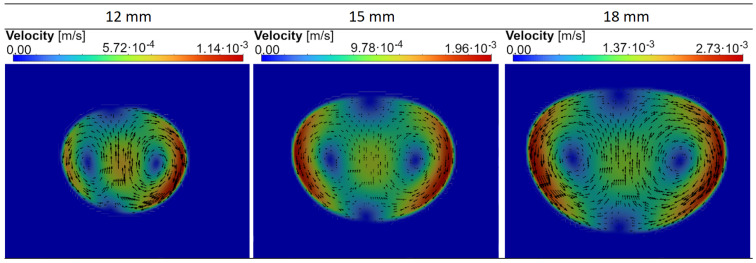
Velocity distribution in the metal after 200 s of the process for three sizes of the load (cross section of a torus with radius: 12, 15 and 18 mm).

Figure 11 shows the velocity distribution in the metal obtained after 200 s of simulation. As the load size increases, the velocity of the liquid metal increases, which is justified by the greater forces acting on the load and the larger volume facilitating the development of flow. One may wonder about the large asymmetry of flow in the case of a small load, decreasing as it increases. This is surprising because the ratio of currents supplying the inner (I1) and outer coil sections (I3) in Table 3 is more or less constant and equal to 1.5. However, the aim is to melt the largest possible metal loads, this phenomenon has no major practical significance. The stirring of metal, forced by the action of electromagnetic forces, is characteristic of all MHD devices for molten metals [45]. It increases the homogenization of the metal during the process and is an important advantage of this group of devices compared to other solutions.

There is some risk that the stability of the metal mass could be disrupted by the stirring effect, potentially causing it to be displaced from the required coil area. However, in the analyzed process, the flow velocity stabilized, preventing this issue from occurring. The likely reason for this positive outcome is that the load is surrounded by a coil, which generates Lorentz forces acting on it from all sides. These forces help prevent deformation on the surface of the load.

**Figure 12:**
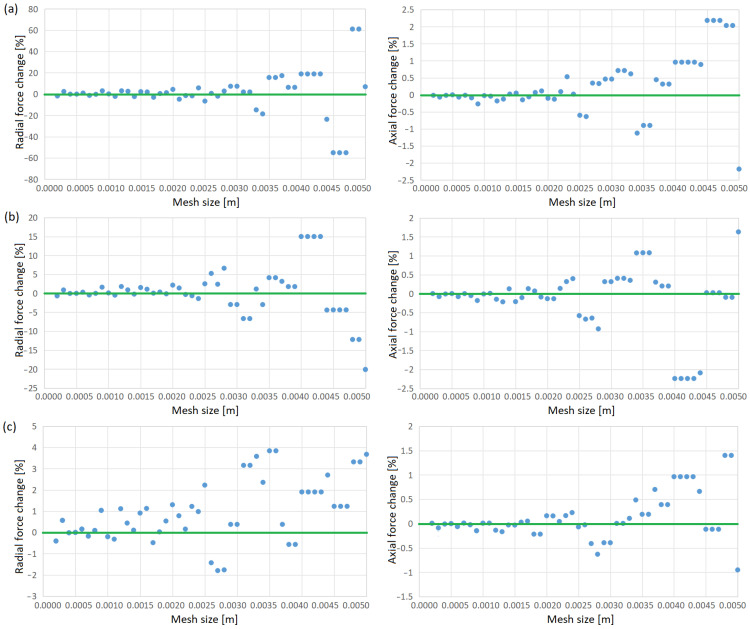
Influence of model mesh density on horizontal and vertical force acting on metal at 200 s: (**a**) 12 mm, (**b**) 15 mm and (**c**) 18 mm. The green line indicates the force value for the 0.005 m grid used in the above tests, with respect to which the change is shown.

In addition to the simulations, a mesh independence test was conducted. Since Lorentz forces are the key factor determining the behavior of the process and the geometry of the liquid metal, the impact of varying the mesh size relative to the 0.0005 m size used in the previous simulations was examined. As shown in Figure 12, a plateau is observed near the applied mesh size (0.0005 m), with only minor fluctuations occurring due to the meshing algorithm. These fluctuations do not exceed approximately 1%. This indicates that the initially selected mesh size is sufficient, and further reduction will not improve the model’s accuracy.

## 4. Conclusions

- This paper presents a novel levitation melting system that utilizes a gutter-shaped coil with four independently powered sections.
- By removing the metal load from the axial region, the proposed system allows Lorentz forces to support the entire lower surface of the metal, in contrast to conventional levitation systems.
- This approach enables the melting of large, industrially relevant metal masses.
- Since the metal remains completely free from contact with the crucible, the proposed device offers advantages over semi-levitation systems with a cold crucible in terms of metal purity, energy efficiency, and the degree of metal overheating.
- Due to the off-axis placement of the metal and the resulting unbalanced forces, maintaining a stable position of the toroidal metal load at the center of the coil channel poses a challenge. Therefore, optimizing the currents supplying the four coil sections was necessary.
- For optimization purposes, a numerical model of the liquid metal process was developed, incorporating bidirectional coupling of electromagnetic and hydrodynamic fields along with the dynamics of the free metal surface.
- The proposed hierarchical system for numerical current optimization ensures a stable position of the analyzed metal masses.
- Numerical experiments have demonstrated that, given the adopted coil geometry, it is possible to stably levitate a titanium load with a mass of 2.6 kg—a sufficient amount of metal for industrial applications.
- A cross-sectional radius of 18 mm for the largest load should maintain the continuity of the toroid during the process, allowing for an uninterrupted current flow around the load and ensuring the stability of the levitation mechanism.
- The developed methodology provides a foundation for constructing a physical device for large-scale electromagnetic levitation. However, this requires the addition of an unconventional power source capable of supplying four different currents, as well as the design of a spiral coil with the proposed 2D cross-section in a real 3D geometry.
- There are no physical obstacles to further scaling the device and the mass of the melted metal. The only limitation is the availability of sufficient power from the mentioned four-channel source.
- When scaling up, the coil geometry can remain unchanged, only the currents need to be re-optimized. However, a potentially interesting further research direction is also the optimization of the coil geometry.
- Although the research was conducted on titanium, the proposed levitation melting system can be used to melt other metals with higher density (e.g., zirconium, niobium, molybdenum, and tantalum). Of course, the optimized current values for these metals necessary to maintain levitation will be higher.

## Figures and Tables

**Table 1:** Comparative analysis of various reactive metal melting methods.

	Vacuum Induction Melting	Classic Levitation	Semi-Levitation (Cold Crucible)	Large-Scale Levitation
**Metal mass**	Very high	Very low	Very low	High
**Metal purity**	Low	Very high	High	Very high
**Metal overheating**	Very high	Very high	Low	Very high
**Energy efficiency**	Very high	High	Low	High

**Table 2:** Properties of pure molten titanium, air, and copper (coil).

Property	Value
Titanium density	4110 kg/m^3^
Electrical conductivity of titanium	0.56 MS/m
Magnetic permeability of titanium	1.00 $\mu_{0}$
Titanium viscosity	4.42 mPa s
Surface tension of titanium	1.56 N/m
Air density	1.225 kg/m^3^
Air viscosity	0.018 mPa s
Magnetic permeability of air	1.00 $\mu_{0}$
Electrical conductivity of copper	58 MS/m
Magnetic permeability of copper	1.00 $\mu_{0}$

**Table 3:** Optimized power supply parameters.

Load Size [mm]	12	15	18
Current 1 [A]	653.5	621.4	573.2
Current 2 [A]	418.8	509.8	589.1
Current 3 [A]	403.3	390.8	366.0
Current 4 [A]	460.8	423.5	374.1

## Data Availability

The raw data supporting the conclusions of this article will be made available by the authors on request.
